# P-708. Risk Factors Associated with Lower Respiratory Tract Infections in Adult Patients with Respiratory Syncytial Virus Infections

**DOI:** 10.1093/ofid/ofae631.904

**Published:** 2025-01-29

**Authors:** Mamta Sharma, Susan M Szpunar, Ashish Bhargava, Louis Saravolatz

**Affiliations:** Henry Ford St John Hospital, Grosse Pointe Woods, MI. 48236, Michigan; Ascension St. John Hospital, MI; Ascension St. John Hospital; Wayne State University School of Medicine, Grosse Pointe Woods, Michigan; Ascension St John Hospital, gross pointe woods, Michigan

## Abstract

**Background:**

Respiratory Syncytial Virus (RSV) is known to cause severe disease in elderly individuals and patients with underlying cardiopulmonary or immunocompromised conditions. Little is known about the factors associated with the lower respiratory tract infections (LRTI).

**Methods:**

A multicenter historical cohort study was conducted on adult patients hospitalized for laboratory-confirmed RSV-related diseases in Ascension hospitals in Southeast Michigan between January 2017 and December 2021. Hospitalized patients were identified using ICD 10 codes for RSV-related diseases. Medical records were reviewed after IRB approval. LRTI was defined as an acute respiratory disorder meeting at least three out of four criteria: respiratory signs/symptoms (cough/ dyspnea/ tachypnea), fever, oxygen saturation below 94%, and abnormal chest x-ray at the time of hospital admission. Data were analyzed using Student’s t-test, the chi-squared test, the Mann-Whitney U test and logistic regression using SPSS v. 29.0.

**Results:**

Of 360 patients, 143 (39.7%) had LRTI. The mean (sd) age of patients with LRTI was 69.1 + 15.3 years, and 76 (53.1%) were female. The mean Charlson Weighted Index of Comorbidity score was 2.5 + 2.1. Factors associated with LRTI in univariable analysis were age, sex, asthma, time period (TP) (pre-COVID (2017-2019) and COVID (2020-2021), maximum temperature within 24 hours of admission (Tmax), lowest diastolic blood pressure, oxygen requirement and neutrophil count at admission, infectious disease (ID) consultation, and antibiotics given for >1 day. Predictors for LRTI in multivariable logistic regression were COVID TP (odds ratios [OR], 2.0; 95% CI 1.2-3.4), Tmax ([OR], 1.2; 95% CI 1.0-1.5), oxygen requirement at presentation (OR, 2.1; 95% CI 1.2-3.4), ID consultation ([OR], 2.7; 95% CI 1.6-4.4), antibiotics given for >1 day (OR, 3.7; 95% CI 2.2-6.2) while female sex was inversely related to LRTI episodes ([OR], 0.4; 95% CI 0.3-0.7) .

**Conclusion:**

Our study finds that COVID time period, maximum temperature within 24 hours of admission, oxygen requirement at admission, ID consultation, and antibiotics given >1 day were significantly associated with LRTI among adult patients with RSV infection. Female sex was less likely to have LRTI. Further studies needed to confirm these findings.

**Disclosures:**

**All Authors**: No reported disclosures

